# Unravelling oncosis: morphological and molecular insights into a unique cell death pathway

**DOI:** 10.3389/fimmu.2024.1450998

**Published:** 2024-08-29

**Authors:** Jie Guo, Wen-tao Yang, Feng-yi Mai, Jing-rong Liang, Jiao Luo, Ming-chao Zhou, Dong-dong Yu, Yu-long Wang, Chen-guang Li

**Affiliations:** ^1^ Department of Rehabilitation Medicine, Shenzhen Second People’s Hospital, Shenzhen, China; ^2^ Guangdong Key Laboratory for Biomedical Measurements and Ultrasound Imaging, National-Regional Key Technology Engineering Laboratory for Medical Ultrasound, School of Biomedical Engineering, Shenzhen University Medical School, Shenzhen, China; ^3^ Pain Department of Huazhong University of Science and Technology Union Shenzhen Hospital (Nanshan Hospital), Shenzhen, China; ^4^ Department of Human Cell Biology and Genetics, Southern University of Science and Technology School of Medicine, Shenzhen, China

**Keywords:** oncosis, cell death, mechanisms, inducer, diseases and therapies

## Abstract

Programmed cell death (PCD) is a fundamental biological process for maintaining cellular equilibrium and regulating development, health, and disease across all living organisms. Among the various types of PCD, apoptosis plays a pivotal role in numerous diseases, notably cancer. Cancer cells frequently develop mechanisms to evade apoptosis, increasing resistance to standard chemotherapy treatments. This resistance has prompted extensive research into alternative mechanisms of programmed cell death. One such pathway is oncosis, characterized by significant energy consumption, cell swelling, dilation of the endoplasmic reticulum, mitochondrial swelling, and nuclear chromatin aggregation. Recent research suggests that oncosis can impact conditions such as chemotherapeutic cardiotoxicity, myocardial ischemic injury, stroke, and cancer, mediated by specific oncosis-related proteins. In this review, we provide a detailed examination of the morphological and molecular features of oncosis and discuss various natural or small molecule compounds that can induce this type of cell death. Additionally, we summarize the current understanding of the molecular mechanisms underlying oncosis and its role in both normal physiology and pathological conditions. These insights aim to illuminate future research directions and propose innovative strategies for leveraging oncosis as a therapeutic tool against human diseases and cancer resistance.

## 1 Introduction

Cell death is a fundamental biological process resulting in the cessation of cellular activity. Historically, it was perceived as an undesirable but inevitable outcome of cellular malfunction, primarily due to the inflammation it invariably triggered following damage. Although critical for tissue formation, maintenance, and repair, cell death can also be initiated by external factors such as injury, environmental stress, or infection, leading to pathological conditions. In recent decades, research has uncovered various forms of genetically programmed cell death (PCD) designed to eliminate unnecessary or severely damaged cells, which can sometimes impact adjacent tissues.

The Nomenclature Committee on Cell Death has established systematic criteria to classify and analyze cell death based on morphological, biochemical, and functional aspects (1). Cell death can be categorized into two primary groups: non-programmed and programmed, depending on whether a specific signal is required to initiate the process (2). Necrosis, a non-programmed form, typically results from irreversible damage caused by severe external factors. Conversely, programmed cell death (PCD) is regulated by physiological or developmental signals (3, 4). PCD is a ubiquitous phenomenon across a broad spectrum of organisms, from microorganisms to higher eukaryotes, and is essential for normal development and maintaining tissue homeostasis through continuous cell renewal. Different forms of cell death, categorized by their morphological and molecular characteristics, include apoptosis and autophagy, which are classified as PCD, and pyroptosis, necroptosis, and NETosis (5), which are classified as inflammatory cell death. Several novel forms of cell death have recently been identified, such as ferroptosis, anoikis, autosis, parthanatos, paraptosis, methuosis, endosis, cornification, mitotic catastrophe, and cuproptosis. These discoveries have expanded our understanding of the complexities of cell death and its regulation (5). These new insights have also enhanced our comprehension of how inflammatory diseases and tumor immunity are managed. Among these, oncosis has gradually increased attention from pathologists as a distinct mode of cell death.

In 1995, through extensive research, Guido Majno differentiated oncosis from apoptosis as a separate mode of cell death. The term “oncosis” is derived from the Greek word “*Onkos*,” meaning cell swelling. This ancient name was coined as early as 1911 and was used by Rudolf von Reckling hausen to describe the death of bone cells accompanied by marked swelling and enlargement of the bony pits and tubules (6). The emergence of cell swelling also reveals a different way of cell death from apoptosis and warrants further investigation. In this review, we provide a comprehensive overview of oncosis, highlighting the morphological and biochemical characteristics of cells undergoing this type of cell death. We discuss recent advancements, including the use of natural or small molecule medicines as oncosis inducers, and explore the potential application of oncosis in cancers and inflammatory disease therapy. Our discussion extends to the molecular mechanisms underlying oncosis and its significance in normal physiology and disease. Most anticancer therapies rely on their ability to induce apoptosis, a process governed by numerous pro-apoptotic genes/proteins. However, resistance to apoptosis, often driven by various factors, poses a significant challenge in cancer treatment compared to other forms of multidrug resistance. Consequently, an alternative approach gaining attention is the induction of non-apoptotic cell death pathways to bypass apoptosis resistance. This review aims to thoroughly assess the strengths and limitations of oncosis, intending to inspire novel strategies to overcome drug resistance in cancer therapy. We hope this review serves as a valuable resource for researchers delving into the intricacies of oncosis, providing insights and further scientific inquiry in this field.

## 2 Relationship between oncosis, apoptosis and necrosis

Apoptosis and oncosis are two primary forms of cell death, each exhibiting distinct morphological and biochemical characteristics (7). Apoptosis is characterized by cellular shrinkage, chromatin condensation and margination, and plasma membrane ruffling commonly referred to as budding. In contrast, oncosis is characterized by cellular swelling, membrane integrity disruption, and intracellular contents release. These released contents are often aggressive and pro-inflammatory, capable of causing significant damage to adjacent tissues (8). Notably, apoptotic cells generally do not induce inflammation because they are typically phagocytosed by immune cells before their intracellular contents can be released (8–10). This discussion emphasizes that although apoptosis and oncosis are mediated by distinct pathways, they overlap in their involvement of cell surface death receptors, mitochondria, and the endoplasmic reticulum. A crucial event in apoptosis is the permeabilization of the mitochondrial outer membrane (MOMP), which triggers the release of cytochrome c, ultimately leading to the activation of caspases. In contrast, oncosis is characterized by the early opening of MPTP (mitochondrial permeability transition pore) in the inner membrane, occurring without the release of cytochrome c. The initiation of MPTP results in rapid loss of mitochondrial membrane potential (ΔΨm), cessation of ATP synthesis, solute influx, and mitochondrial swelling (11).

Mitochondria play a central role in oncotic cell death due to their involvement in ATP production and reactive oxygen species (ROS) synthesis (12). In oncosis, ROS contributes to cellular damage by disrupting cellular structures and functions. Excessive ROS production leads to mitochondrial dysfunction, ATP depletion, and the failure of ion pumps, which are critical for maintaining cellular homeostasis. ROS causes mitochondrial membrane permeabilization, leading to the loss of ΔΨm and further ROS generation (13). Oncosis results in necrotic cell death characterized by cell swelling, membrane rupture, and inflammation due to the release of intracellular contents into the extracellular space (14). It is noteworthy that a recently identified ROS-sensitive cell death pathway, designated as oxeiptosis, further emphasizes the pivotal role of ROS in cellular processes. This pathway is specifically triggered by elevated levels of ROS, particularly hydrogen peroxide (H_2_O_2_), and serves as a protective mechanism to mitigate damage caused by oxidative stress. The KEAP1-PGAM5-AIFM1 pathway plays a pivotal role in the process of oxeiptosis. KEAP1 detects ROS and activates PGAM5, which in turn dephosphorylates AIFM1 at Ser116. This dephosphorylation is crucial for the execution of oxeiptosis (15). Unlike oncosis, oxeiptosis is a non-inflammatory form of cell death. It prevents excessive immune responses and inflammation, which can be detrimental to surrounding tissues. Oxeiptosis leads to a controlled form of cell death that does not involve caspases and avoids triggering inflammation. This makes it a unique and beneficial response to oxidative stress, particularly in conditions where inflammation needs to be minimized (15). The level of ATP within a cell is crucial for determining whether apoptosis or oncosis occurs. Apoptosis, an energy-dependent process, requires ATP to initiate and sustain the molecular events leading to cell death. In contrast, oncosis is associated with significant depletion of ATP (16). Despite these the distinct differences, both oncosis and apoptosis can occur simultaneously within the same lesion site. For example, in the pathological changes of acute viral hepatitis, one cell type death is characterized by edema, vacuolation and ballooning of liver cells, and then develops into a morphological change of dissolving necrosis, indicative of oncosis-like cell death. The other involves eosinophilic degeneration of liver cells, characterized by nuclear concentration and disappearance, resulting in the formation of eosinophilic bodies, also known as apoptosis-like necrosis. Similar phenomena have been observed in intraductal carcinoma of the breast with acne-like substances and myocardial infarction lesions (17). Simultaneously, these two types of cell death exhibit distinct distribution patterns: apoptosis is more frequent in regions with adequate blood supply, while oncosis is more prevalent in areas with insufficient blood supply. Apoptosis is an active energy-consuming process, and its completion requires ATP. Oncosis is a passive process with minimal or no energy consumption. Under the identical stimuli, whether the cells undergo apoptosis or oncosis depends on their ATP content. When ATP is deficient, apoptosis cannot be completed and cells resort to oncosis. Supplementation with ATP can facilitate the transformation of oncosis into apoptosis (18) (**Table 1**).

**Table 1:** Characteristic features of apoptosis, oncosis and necrosis.

Characteristics	Apoptosis	Oncosis	Necrosis
Cell size	reduced (shrinkage)	increased (swelling)	increased (swelling)
Plasma membrane	intact	intact in the early phase and disrupted in the late phase	disrupted membrane
Nucleus	fragmentation of DNA into nucleosome; nuclear chromatin condensation	nucleus dilatation and clumping of chromatine reticular nucleolus	late random digestion of DNA
Specific features	apoptotic bodies	membrane blebbing; organelles swelling	cytoplasm released; organelle disintegration
Energy balance	retained ATP production	ATP depletion;	not dependent on energy;
Caspase dependent	dependent on caspase family members (caspase3/6/7/8/9)	independent on caspase family members	independent on caspase family members
Adjacent inflammation	rare	frequent	frequent

Distinguishing between oncosis and apoptosis is critical for testing new therapeutic drugs, as both forms of cell death can be triggered by the same drug. It is also critical to dynamically assess the predominant effect of a drug at various concentrations (19). Numerous dose-response studies have shown that cells exposed to low concentrations of drugs like cisplatin, arsenic trioxide, etoposide, or doxorubicin exhibit apoptotic characteristics, while higher doses lead to oncotic features (20). Tumor cells have a minimal impact on surrounding tissues following apoptosis. Conversely, oncosis can cause destruction of the cell membrane, leading to the release of cell contents and triggering inflammatory reactions in surrounding tissues. Therefore, our future research will focus on guiding the transformation from oncosis to apoptosis and stabilizing the cell membrane. Achieving stable cell membrane and reasonable guidance of the transformation from oncosis to apoptosis could allow for a higher dosage of drugs without increasing their toxicity, thereby enhancing anti-tumor efficacy.

Unlike apoptosis, necrosis represents an alternative, uncontrolled form of cell death that is induced by external injury, such as hypoxia or inflammation. This process frequently entails the upregulation of various pro-inflammatory proteins and compounds, such as NF-кB resulting in the rupture of the cell membrane causing spillage of the cell contents into surrounding areas. This release triggers a cascade of inflammation and tissue damage (7, 21). In contrast to apoptosis, necrosis is an energy independent form of cell death, where the cell is damaged so severely by a sudden shock (radiation, chemicals, hypoxia *etc*) that it is unable to function. As oncosis progresses, there is a depletion of intracellular energy stores and an eventual failure in the ionic pumps of the plasma membrane. The final outcome of oncosis is necrosis, which is referred to as oncotic necrosis. It should be noted that a cell undergoing apoptosis may exhaust its ATP supply, rendering it unable to complete apoptotic process. This can result in secondary necrosis, which is also characterized by swelling and lysis. Conversely, if oncosis is inhibited, cellular stress may induce apoptosis. This switch from apoptosis to secondary necrosis, via oncosis shows that apoptosis and oncotic necrosis are closely related phenomenon. Therefore oncosis could act as a link between apoptosis and necrosis.

## 3 Morphological characteristics of oncosis

Different forms of cell death have different morphological characteristics. Oncosis stands out as a form of cell death distinct from apoptosis, with several key differences. Under an optical microscope, oncosis was characterized by swollen cells, demonstrating an increase in cell volume. The cytoplasm becomes looser, showing dense granules and a swollen endoplasmic reticulum. The nucleus is swollen, and the chromatin in the nucleus is scattered, agglutinating around the nuclear membrane and nucleoli. This chromatin sometimes condenses into a mass and often shows nucleolysis in the later stage. The cell membrane exhibits a bubble-like protrusion, with the generated bubble comprising primarily liquid and rarely containing organelles. As the permeability of the cell membrane increases, its integrity is compromised and eventually disintegrates. The cellular contents then overflow, inducing autolysis-like changes and triggering pronounced inflammatory reactions around cells. Oncosis can be caused by various factors, resulting in morphological changes such as cell water denaturation, balloon degeneration, dissolution and necrosis (7). Electron microscopy reveals that organelles in swollen cells, particularly mitochondria, exhibit swelling. mitochondria swelled, cristae were destroyed, flocculent changes or high dense particles appeared in the later stage; chromatin initially agglutinates, followed by DNA fragmentation (22). It has been reported that the microvilli on the cell membrane disappear, the integrity is destroyed, and pores appear in the early stages, which results in an increased permeability of the cell membrane (7). These distinct morphological features of oncotic cells provide significant value in the diagnosis of swollen dead cells. However, relying solely on morphological has certain limitations and may not conclusively identify the mode of cell death. Therefore, it is necessary to corroborate these results by integrating the morphological data with other detection methods.

## 4 Cellular mechanism of oncosis

Current understanding of the molecular mechanisms underlying oncosis is based on four main mechanisms. The initial mechanism of oncosis involves damage to cell membranes. It is widely accepted that the occurrence of oncosis is primarily attributable to alterations in the cell membrane integrity. The dysfunction of cell membranes, whether due to physiological or pathological factors, can result in the physical defects of cell membranes and the subsequent onset of oncosis. Early in oncosis, changes in membrane permeability occur, which is a key factor in determining this mode of cell death. Schlossman (23) proposed that oncosis begins with cell membrane injury, influenced by multiple factors including direct destruction by injury agents, elevated Ca^2+^ levels, the activation of phosphoesterase A2, and so forth (22). Recent research has successfully cloned a membrane-specific receptor from the cell membrane, known as porimin. This receptor, composed of 118 amino acids and part of the transmembrane mucin family, regulates membrane swelling and cell death, mediating oncotic cell death prior to membrane injury. Upon binding with its ligands, porimin rapidly facilitates the formation of pores in the cell membrane, increasing permeability and leading to severe cellular damage, a hallmark of oncosis (23).

The second mechanism is the inhibition of ATP synthesis. A significant reduction in intracellular ATP levels represents a pivotal biochemical mechanism in the induction of oncosis. The reduction of ATP disrupts the function of Na^+^-K^+^-ATPase ion pump and Ca^2+^-ATPase ion pump on the cell membrane, which leads to the increase of sodium and chloride ion concentrations and water retention. This imbalance results in cell swelling and increased Ca^2+^ concentration (24). The reduction in aerobic metabolism and the elevation of glycolysis result in the accumulation of intracellular lactic acid. The rapid decline in ATP levels accelerates the onset of oncosis (24).

The third mechanism involves the opening of MPTP, which results in mitochondrial dysfunction. MPTP activation occurs in response to cellular stress and death signals, regulating mitochondrial membrane permeability. This activation causes significant swelling of the mitochondria, impaired ATP synthesis, and an accumulation of Ca^2+^. According to the open theory of MPTP, the accumulation of ROS in the mitochondria and its subsequent oxidation events contribute to membrane damage. The opening of MPTP pores facilities the massive release of cytochrome c, a pivotal event that significantly reduces ATP level, and precludes adequate time for caspase activation. This rapid sequence of events precipitates extensive cellular swelling and cell death. Bcl-XL, an anti-apoptotic protein, plays a significant role in maintaining mitochondrial integrity and regulating bioenergetics, which is crucial for cell survival. By interacting directly with the β-subunit of ATP synthase, Bcl-XL reduces proton leakage and enhances ATP synthesis efficiency. Research indicates that over-expression of Bcl-XL in cells can maintain intracellular ATP, thus inhibiting aspirin-induced oncosis (25). This is particularly important in pathological conditions where cell survival is compromised. Bcl-XL also inhibits the release of cytochrome c from the mitochondria by preventing MOMP. By inhibiting cytochrome c release, Bcl-XL prevents the formation of the apoptosome and the subsequent activation of caspases, thus promoting cell survival. This inhibition is crucial not only in apoptosis but also impacts oncosis by maintaining mitochondrial integrity and preventing the early release of cytochrome c, which might otherwise contribute to cell death processes. Furthermore, Bcl-XL can directly bind to cytochrome c, sequester it, and prevent its participation in apoptotic signaling pathways. This binding may also play a role in oncosis by modulating the availability of cytochrome c for other cellular processes, potentially delaying or altering the course of cell death. The reduction in ATP levels impedes the progression of apoptosis, thereby shifting the cell towards oncosis (26). Conversely, F_1_F_0_-ATP synthase (ATP synthase), a vital enzyme within the mitochondrial inner membrane, synthesizes ATP through oxidative phosphorylation. In addition, calpain (a Ca^2+^-dependent proteolytic enzyme) has been demonstrated to promote the occurrence of oncosis. Conversely, the calpain inhibitor PD150606 has been shown to inhibit oncosis, highlighting the complex interplay of molecular pathways that govern this type of cell death (27).

**Figure 1:**
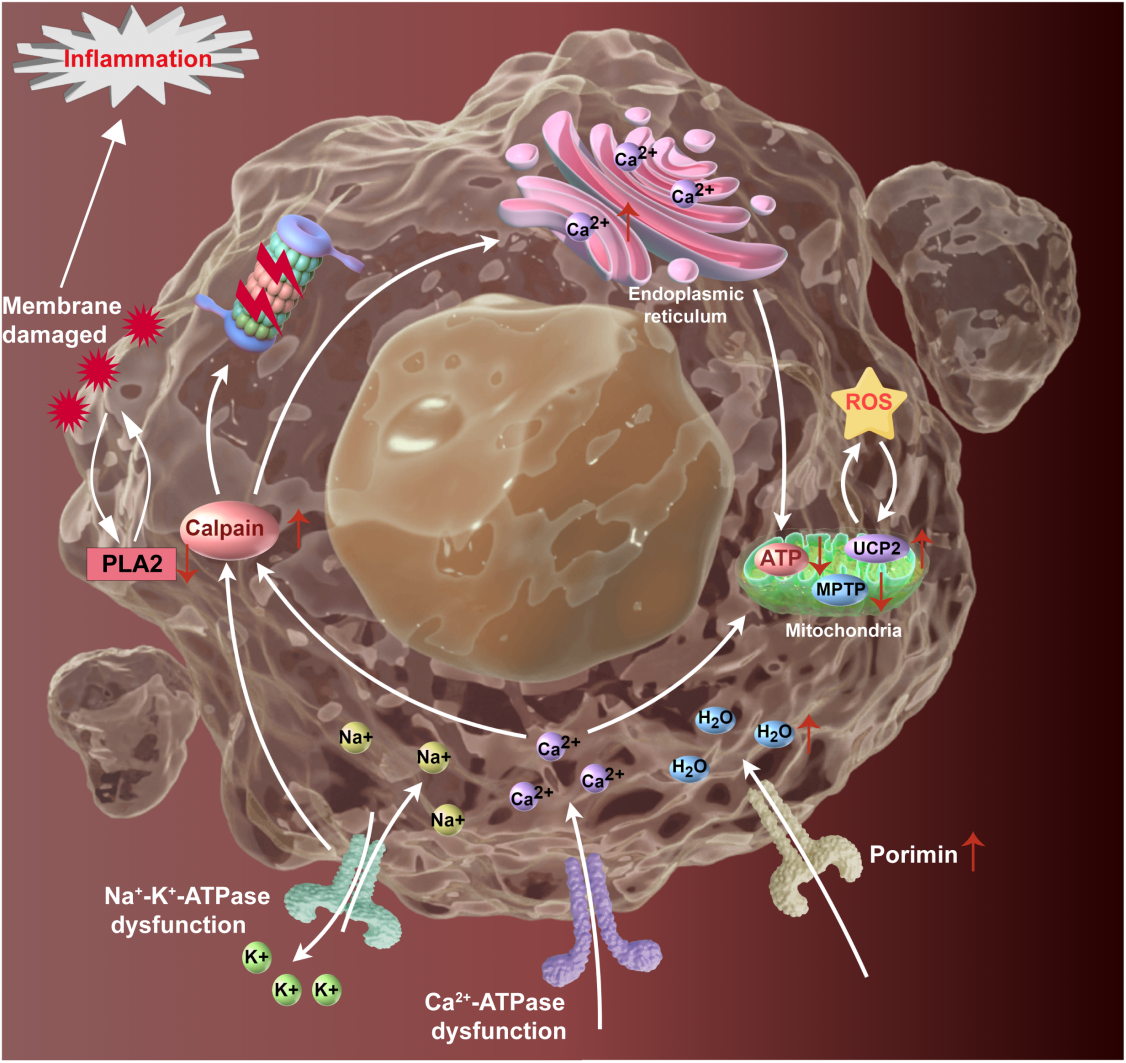
The molecular mechanism of oncosis is graphically illustrated. After treatment with oncosis inducers, oncosis is triggered by inhibition of ATP synthesis, mitochondrial dysfunction, disruption of the ion balance and damage cell membrane. This results in the cells undergoing swelling. Ultimately, the further entry of water molecules results in the rupture of the cell membrane and the subsequent release of intracellular contents, which induces inflammation. ATP, adenosine triphosphate; MPTP, mitochondrial permeability transition pore; UCP2, uncoupling protein 2; ROS, reactive oxygen species; PLA2, phospholipase A2.

The fourth mechanism is ion balance disorder, which can trigger oncosis. Ion transport plays a crucial role in maintaining the transmembrane osmotic gradient. Cells sustain a physiological osmotic balance through the activity of the electrogenic Na^+^-K^+^-ATPase pump (which moves 3 Na^+^ out and 2 K^+^ in), creating an intracellular environment high in potassium (around 140mM) and low in sodium (approximately 10mM). This pump uses ATP as the driving force to transport Na^+^ and K^+^ across the cell membrane (28). During oncosis, ATP synthesis is inhibited, which causes the dysfunction and deactivation of Na^+^-K^+^-ATPase and Ca^2+^-ATPase ion pumps. This disruption results in an elevation of sodium and chloride ion concentrations, accompanied by an increase in water retention, which in turn leads to cellular swelling and a rise in Ca^2+^ concentration. It is suggested that Ca^2+^ play a role in regulating cell volume and death. After oncosis, the concentration of intracellular Ca^2+^ increases in certain cell types. This increase occurs either through enhanced influx of Ca^2+^ across the sarcolemma or release from intracellular stores (29). Excessive Ca^2+^ concentration is detrimental, leading to extensive cell damage. The increase of intracellular Ca^2+^ concentration is partly due to the overload of Ca^2+^ intake by mitochondria contributes to mitochondrial dysfunction and reduced ATP synthesis. Concurrently, elevated Ca^2+^ stimulate the activity of a multitude of Ca^2+^-dependent degradation enzymes, including phospholipases and calpains, which degrade membrane phospholipids and compromise cell membrane integrity. Although elevated Ca^2+^ may not directly regulate cell volume, it can affect cytoskeletal elements such as actin filaments (30). Eventually, ion imbalance resulted in alterations to the cell volume and skeleton, culminating in cell death (**Figure 1**).

In the initial stages of oncosis, cells exhibit a discernible swelling. As the permeability of the cell membrane increases further, cells begin to bubble and lose the integrity of the membrane. At this stages, non-membrane permeable dyes such as propidium iodide can enter the cell (as an indicator of cell death), endoplasmic reticulum and mitochondria begin to swell, chromatin agglutinate, and cell membrane appears vacuolation. The final outcome of oncosis is the complete collapse of the cell membrane. At this stage, the cell enters the stage of necrosis, with the endoplasmic reticulum and mitochondria becoming swollen, ribosomes being lost, lysosomes becoming swollen and rupturing, and the nucleus condensing and dissolving. At the terminal stage, the cell dies, and releases its contents, such as damage-associated molecular patterns (DAMPs), including lactate dehydrogenase (LDH) and high mobility group protein B1 (HMGB1), and eventually inducing an inflammatory response. This event also one of the key distinctions between oncosis and apoptosis.

## 5 Oncosis associated proteins

### 5.1 Porimin

The porimin (pro-oncosis receptor inducing membrane injury) protein, is a member of the membrane-associated mucin family and is uniquely expressed on the surface of cells undergoing oncosis (23). When porimin forms a complex with its ligand, it rapidly compromises the integrity of the cell membrane, leading to pore formation and increased membrane permeability, thereby initiating the process of oncosis. As a glycosylated protein, porimin belongs to the cell membrane-associated mucin family. In 1998, Ma et al. cloned the cDNA of porimin and discovered that it encodes a membrane mucin that induces a type of cell death in Jurkat cells similar to oncosis. Expression of porimin cDNA in COS7 cells resulted in new morphological changes, causing cell membrane damage and cell death upon administration of an anti-porimin antibody. Further studies have demonstrated that some oncosis inducers, such as dihydrotanshinone (31) or cyclometalated iridium(III) complexes (32), can provoke porimin-dependent oncosis through ROS-mediated mitochondrial dysfunction in NSCLC. Despite these insights, the detailed molecular and biochemical mechanisms driving oncosis remain largely unexplored. Further molecular cloning of receptors mediating oncosis-like cell death could provide deeper insights into this novel pathway of non-apoptotic cell death.

### 5.2 Uncoupling protein 2

Severe metabolic insults, such as ischemia, lead to significant energy depletion in cells, causing a substantial decrease in mitochondrial respiration and ATP production. This energy deficit disrupts ionic homeostasis and results in cellular swelling. The generation of ATP in mitochondria depends on an electrochemical gradient, and a decrease in ΔΨm often precedes the morphological changes observed in oncotic cell death (33). The uncoupling protein (UCP) family is essential in regulating ΔΨm. These proteins are found in the inner mitochondrial membrane, where they balance transmembrane proton concentration, mitigate oxidative phosphorylation, and inhibit ATP synthesis (34). UCP-2, in particular, is broadly expressed across various tissues and plays a key role in controlling ΔΨm (35). *In vitro* studies indicated that higher UCP-2 expression lowered the ΔΨm and reduced ATP production, leading to cell death with features resembling oncosis. Conversely, the absence of UCP-2 results in an increased ΔΨm and higher ATP levels, likely counteracting the reduced energy production caused by UCP-2’s uncoupling function. Therefore, blocking these uncoupling proteins might prevent oncotic cell death in hypoxic environments by maintaining ΔΨm and sustaining ATP production (33). However, the role of UCP-2 in ischemic stroke remains contentious. Studies *in vivo* involving UCP-2 knockout mice have shown an unexpected increase in infarct size, linked to decreased expression of anti-oxidant, cell-cycle, and DNA-repair genes, and elevated levels of inflammatory mediators (36). UCP-2’s neuroprotective effect is believed to stem from its antioxidant properties, which help reduce oxidative stress during cerebral ischemia-reperfusion. In models of cerebral ischemia-reperfusion, mice overexpressing UCP-2 exhibit less neuronal damage and lower ROS levels (37). Nonetheless, the exact function of UCP-2 in oncosis is not yet fully understood, necessitating further research on oncosis will improve our understanding of this important biological protein.

### 5.3 Calpain

To date, 14 members of the calpain protease family have been identified in mammals (38). Calpains are categorized based on their tissue distribution into ubiquitous or tissue-specific isoforms (39). Notably, µ-calpain and m-calpain are activated by different Ca^2+^ concentrations *in vitro*, with µ-calpain requiring micromolar (µM) levels and m-calpain necessitating millimolar (mM) levels of Ca^2+^ (40, 41). Miyoshi et al. (42)proposed that µ-calpain activation contributes to oxidant-induced death in hepatocytes. In contrast, Liu et al. found that both µ- and m-calpain are expressed in rabbit renal proximal tubular cells and that calpain inhibitors could not distinctly implicate either isoform in oncosis (43). Similarly, Blomgren et al. proposed that m-calpain activation is involved in the pathophysiology of hypoxic/ischemic brain injury (44). Consequently, there is no clear consensus on the specific roles of µ- and m-calpain in oncosis. Since most cell types express both isoforms and the available calpain inhibitors are not isoform-specific in cellular or *in vivo* contexts, molecular approaches such as inducible gene-knockout animal models, isoform-specific antisense oligonucleotides, or small interfering RNA technologies are needed to clarify their distinct roles in oncosis.

Increasing evidence suggests that calpains, a family of Ca^2+^-activated proteases, play crucial roles in various cellular processes, including mitochondrial dysfunction and the collapse of the cytoskeleton. These neutral cysteine proteases are considered central players in the process of oncotic cell death due to their ability to degrade cytoskeletal proteins, leading to the breakdown of the cytoskeleton (45). Activation of calpains contributes significantly to mitochondrial dysfunction and the degradation of both the cytoskeleton and the plasma membrane. Several cellular factors can enhance calpain activity: increases in cytosolic Ca^2+^ concentration, translocation of calpains to membranes, autolysis of pro-calpains, dissociation of calpain subunits, reduced levels of calpastatin (the endogenous calpain inhibitor), and interactions with calpain activator proteins or phospholipids (46). The balance and interaction of these factors are crucial for regulating calpain activity. However, the specific calpain isoforms involved, their activation mechanisms, intracellular targets, and the pathways by which calpain-mediated proteolysis leads to cell death require further investigation to be fully understood.

### 5.4 Phospholipase A2

Phospholipase A2s (PLA2s) constitute a superfamily of esterases that hydrolyze the *sn*-2 ester bond in phospholipids, releasing free fatty acids and lysophospholipids. PLA2s are pivotal in mediating inflammatory responses and signaling various cellular processes. PLA2s are categorized into three isoforms: secreted (sPLA2), Ca^2+^-dependent cytosolic (cPLA2), and Ca^2+^-independent (iPLA2) (47). Despite over two decades of research on PLA2s’ role in oncosis, much remains unclear. Initially, it was theorized that PLA2 activity increases during oncosis, accelerating the hydrolysis of membrane phospholipids and leading to heightened plasma membrane permeability and cell lysis (48). Experiments have demonstrated that the cell lysis following lethal factor stimulus was reduced by PLA2s inhibitors such as mepacrine or dibucaine. Regrettably, in numerous instances, the investigators failed to document any increase in PLA2 activity or confirm the effectiveness of PLA2 inhibitors in inhibiting PLA2. Nevertheless, studies have indicated that dibucaine and mepacrine diminish the toxicity induced by tert-butyl hydroperoxide, a common oxidative stress model, correlating this reduction with inhibited arachidonic acid release (49). Although specific PLA2 inhibitors were not used in these studies, it was concluded that PLA2’s role in renal cell oncosis is largely stimulus-dependent. The development of more selective inhibitors has furthered our understanding of PLA2s’ involvement in oncosis, emphasizing that their contribution depends on the specific PLA2 isoform (47). For instance, Kohjimoto et al (50) showed that pre-treating cells with the cPLA2 inhibitor AACOCF3 significantly reduced oxalate toxicity by inhibiting arachidonic acid release before cell death. This effect was attributed to the inhibition of arachidonic acid release prior to cell death, which was specifically blocked by the cPLA2 inhibitor AACOCF3, but not inhibitors of sPLA2 and iPLA2. These findings suggest that PLA2s inhibitors and measured PLA2 activity to propose that cPLA2 plays a role in renal cell oncosis. Additionally, Sapirstein et al. (51)found that overexpression of cPLA2 in kidney epithelial cells increased susceptibility to H_2_O_2_ toxicity, whereas overexpression of sPLA2 did not produce the same effect. These findings indicate that cPLA2 is implicated in oxidant-induced oncosis. However, the precise mechanisms by which cPLA2 mediates cellular injury are not yet fully understood. It is also possible that the release of membrane phospholipids due to oxidative lipid peroxidation or PLA2 metabolism may compromise membrane integrity independently of free fatty acids or lysophospholipids. Free fatty acids and lysophospholipids might serve as precursors for bioactive metabolites, promoting inflammation and potentially increasing PLA2 activity. Alternatively, elevated cytosolic-free Ca^2+^ may also increase cPLA2 activity, potentially in conjunction with released membrane phospholipids. Overall, increased cPLA2 activity appears to exacerbate toxicity, with most studies highlighting a central role for Ca^2+^ in facilitating oncosis.

### 5.5 PARP

Poly (ADP-ribose) polymerase (PARP) plays a critical role in the post-translational modification of proteins in response to a wide range of endogenous and environmental genotoxic agents. PARP is crucial for regulating various cellular processes, including DNA repair, cell death, chromatin dynamics, and genomic stability. Although the involvement of PARP in cell death has been postulated for several years, recent research has solidified its role as a pivotal mediator of both apoptosis and necrosis. PARP was the first cellular protein discovered to undergo specific cleavage during apoptosis (52). This cleavage occurs universally in apoptosis induced by numerous stimuli (53). During apoptosis, PARP is cleaved at the DNA binding domain, precisely at the conserved ^211^DEVD^214^ site between Asp^214^ and Gly^215^, resulting in the production of two polypeptides with masses of 24 and 89 kDa. This cleavage effectively inactivates PARP’s catalytic function. Activation of PARP, as a consequence of oxidant-induced DNA strand scission, results in the depletion of cellular NAD^+^ and an alteration of the energy charge ratio, which ultimately forces the cell into oncosis (54, 55). In cases of oxidant-induced DNA damage, current research suggests that PARP inactivation is necessary for apoptosis to proceed. Furthermore, studies have shown that inhibiting PARP can switch oxidant-induced oncosis to apoptosis in thymocytes (56). Thus, PARP activity is a crucial factor in determining the pathway of cell death induced by oxidative stress.

### 5.6 Na^+^/K^+^ -ATPase

The Na^+^/K^+^-ATPase, part of the P-type ATPase protein family, is present in all mammalian cell membranes. Its primary function is to maintain the resting membrane potential by actively transporting three Na^+^ ions out of the cell in exchange for two K^+^ ions into the cell from the extracellular space (57). This process is driven by ATP hydrolysis, making the pump highly sensitive to ATP depletion (58). It has been reported that ischaemic stroke leads to a reductionin oxygen and ATP, resulting in pump failure. Consequently, this causes a rapid rise in cytosolic Na^+^ and Ca^2+^ concentrations coupled with a decline in cytosolic K^+^. Consequently, the malfunction of the Na^+^/K^+^-ATPase pump significantly contributes to cellular oncosis (59). It has been suggested that pharmacological inhibition of the Na^+^/K^+^-ATPase mimics ischemic pump failure, resulting in uncontrolled ionic influx and oncosis. Contrary to this view, recent findings indicate that inhibiting Na^+^/K^+^-ATPase can actually reduce cell death by preserving ATP and preventing autophagy (59). This challenges the traditional understanding of the Na^+^/K^+^-ATPase pump’s pathological role in ionic dysregulation and oncosis. Further investigation is necessary to clarify the Na^+^/K^+^-ATPase’s role in the context of stroke. In cases of hepatic ischemia-reperfusion injury, Na^+^/K^+^-ATPase activity is notably reduced compared to control conditions, and the proportion of oncotic cells increases significantly. Pre-treatment with sulforaphane, an Nrf2 activator, has been shown to restore Na^+^/K^+^-ATPase activity and decrease the extent of cell oncosis following hepatic ischemia-reperfusion, suggesting a protective effect against such injury (60).

## 6 Oncosis-related genes

**Figure 2:**
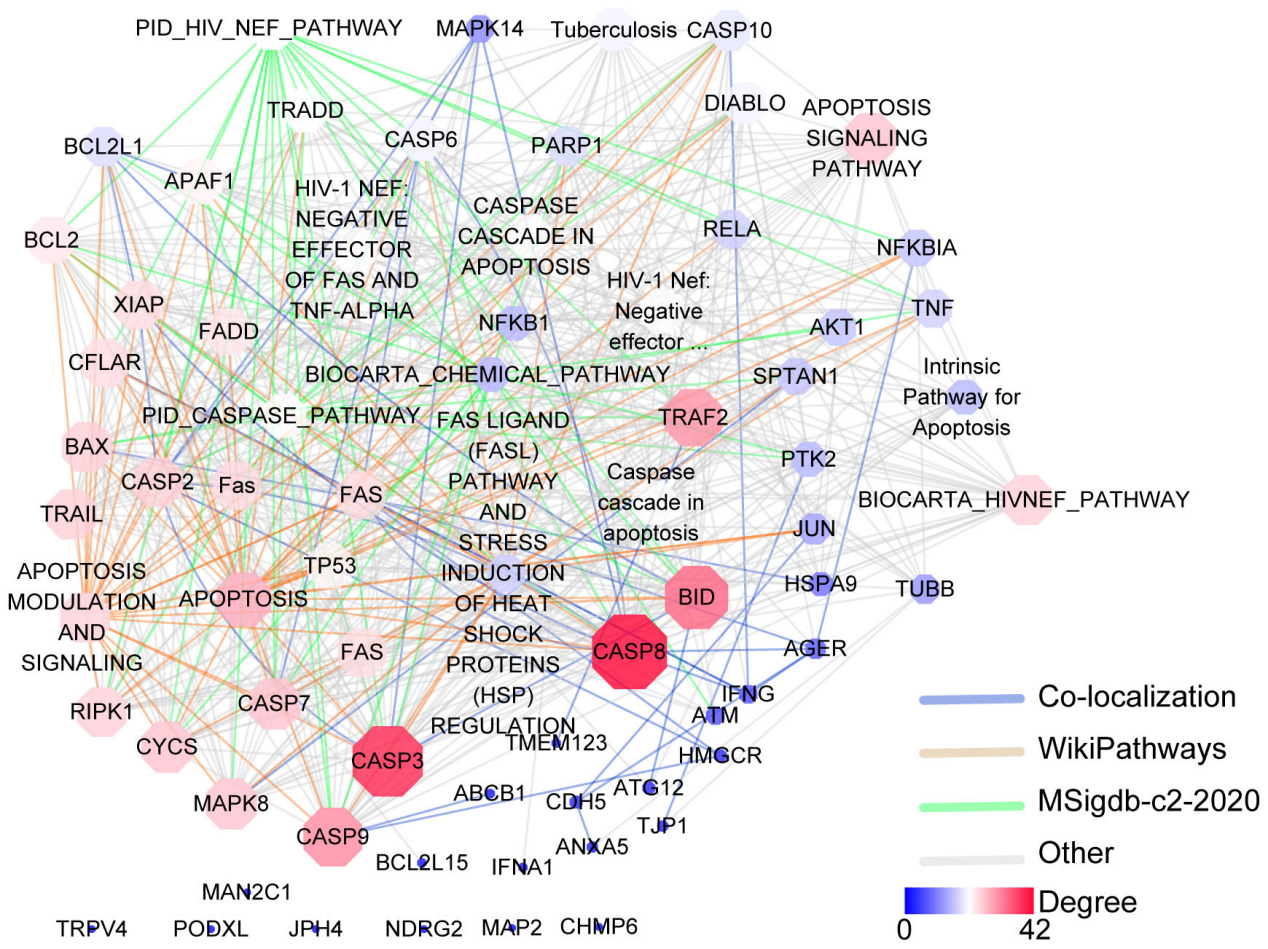
*Gene*–*gene* interaction analysis for the oncosis using the GENEMANIA database. Oncosis key related-genes and associated annotated databases are illustrated in the network legend. Nodes are color-coded and sized according to their degree of connectivity. Nodes with a higher degree of connectivity are shown in red shape, and nodes with a lower degree are depicted in blue shape.

A plethora of genes are implicated in the processes of oncosis, with their expression regulating its occurrence and progression. By utilizing the GeneCards database (https://www.genecards.org/), a query with the keyword “Oncosis” resulted in the retrieval of data integration from 125 databases, identifying 34 genes related to oncosis. Concurrently, an interaction network diagram of the 34 genes was constructed using the online GENEMANIA (http://genemania.org/) analysis tool (**Figure 2**). It is beneficial to comprehend the underlying mechanisms of oncosis by closely examining the alterations in these related genes when investigating oncosis.

## 7 Oncosis and disease

Recent studies have reported an increasing incidence of oncosis, observed under various conditions. For example, oncosis has been noted in the killing of human monocyte-derived macrophages by the virulent *Shigella flexneri (* 61), in the destruction of neutrophils and macrophages by *Pseudomonas aeruginosa (* 62), and in the elimination of macrophages by different strains of *Escherichia coli*. Additionally, it is seen in cell death caused by *Bacillus anthracis* toxin (63), radiation exposure, and ischemic conditions such as heart disease and stroke (64). Emerging evidence suggests that oncosis serves as a bacterial survival mechanism, enabling pathogens to evade the host immune system and establish colonization in host tissues. Understanding oncosis, a process somewhat similar to apoptosis, is crucial for advancing our knowledge of disease mechanisms. This understanding is especially significant in the context of combating cancer, cardiovascular diseases, stroke, AIDS, and various infections. Molecular insights into oncosis can also shed light on how anti-cancer drugs work, potentially aiding in the identification of new therapeutic targets to enhance treatment efficacy.

### 7.1 Oncosis in chemotherapeutic cardiotoxicity

Chemotherapy-induced cell injury has become a growing concern among cancer survivors, significantly impacting the burgeoning field of cardio-oncology. Among various chemotherapeutic drugs, anthracyclines such as doxorubicin are widely recognized for their efficacy in treating both solid and hematological tumors. However, the use of doxorubicin is limited by its cumulative cardiotoxic side effects, which are dose-dependent, progressive, and irreversible (65). Doxorubicin therapy is associated with several cardiotoxic complications, including heart failure, myocardial ischemia, hypertension, thromboembolism, and arrhythmias (66). Literature reveals that doxorubicin induces both apoptosis and oncosis in cardiomyocytes with apoptosis occurring at lower concentrations and a characteristic morphology of oncosis at higher concentrations (67). This suggests that doxorubicin-induced cardiotoxicity may result from cardiomyocyte damage through both apoptotic and oncotic pathways (67).

In addition to doxorubicin, other chemotherapeutic agents like arsenic trioxide, cisplatin, and etoposide have been shown to induce both oncosis and apoptosis (19). Recent research reveals that apoptotic and oncotic mechanisms can coexist, creating a hybrid form of cell death where oncotic features dominate in the final stages of irreversible injury (68). Understanding these pathways is crucial for diagnostic cardiac pathology and developing mechanism-based treatments for chemotherapeutic cardiotoxicity.

### 7.2 Oncosis in myocardial ischemic injury and infarction

Extensive studies have demonstrated that severe myocardial ischemia following coronary artery occlusion causes a specific pattern of damage in cardiomyocytes, marked by mild cell swelling and progressive membrane deterioration, a process known as oncosis (69). Upon reperfusion, this can escalate into significant cell swelling. Recently, apoptosis has also been identified in myocardial ischemia and reperfusion models (68). Studies utilizing caspase inhibitors and mutant mouse models have shown reduced infarction size, indicating that both oncosis and apoptosis contribute equally to early myocardial ischemia-induced cell death (68). Cardiomyocytes may experience both oncosis and apoptosis, depending on the extent and rate of ATP depletion. Rapid ATP depletion tends to promote oncosis, whereas partial ATP preservation leans towards apoptosis (70). Consequently, the injury and cell death caused by a substantial reduction in blood flow can be viewed as a hybrid pattern, termed ischemic cell injury and cell death.

### 7.3 Oncosis in stroke

Oncosis is a crucial mechanism of cell death in ischemic stroke, affecting various central nervous system (CNS) cells, including neurons, glial cells, and vascular endothelial cells (71). During a stroke, energy depletion leads to the failure of ionic pumps and disruption of ionic equilibrium. The resulting imbalance between the influx of Na^+^ and Cl^-^ ions and the efflux of K^+^ ions through channel proteins and transporters creates a transmembrane osmotic gradient, causing water to flow into the cells—a process termed oncosis, which is marked by cell swelling. Oncosis significantly contributes to cerebral edema in ischemic stroke. It directly influences cytotoxic edema and indirectly contributes to vasogenic edema by causing vascular endothelial cell death and compromising the blood-brain barrier. Therefore, targeting the prevention of uncontrolled ionic flux could be a promising strategy for neuroprotection in stroke.

Research indicates that all brain cell types, including neurons, glial cells, and vascular cells, can undergo oncosis following a stroke. Buja et al. have outlined the cellular changes in oncosis, categorizing them into three stages (71). Initially, in stage one, ionic pumps fail to maintain ionic balance across the cell membrane due to ATP depletion, leading to cell swelling as water and ions enter the cell (72). In the second stage, irreversible damage to the cell membrane permits the entry of larger molecules, such as propidium iodide (PI) and trypan blue. The third stage is characterized by the physical rupture of the cell membrane, signifying the transition to necrosis. This final stage is common across various forms of cell death. Considering the involvement of multiple ions and their transporters, an effective strategy would be to target several pathways concurrently, achieving a synergistic effect in preventing oncotic cell death. Inhibiting oncosis could alleviate both cytotoxic and vasogenic edema. Stabilizing the blood-brain barrier (BBB) would likely reduce subsequent neuroinflammation. As drug discovery advances, targeting oncosis could become a potent approach for neuroprotection after a stroke.

### 7.4 Oncosis in cancer

Cell death serves as a crucial physiological mechanism for regulating cell proliferation, stress responses, and maintaining homeostasis. It also acts as a tumor-suppressing mechanism. Numerous strategies have been identified that allow tumor cells to evade or restrict the cell death pathways that normally protect healthy cells (73). Under certain conditions, such as cancer treatment, cell death is undoubtedly beneficial. Known forms of cell death include necrosis, apoptosis, necroptosis, autophagy, anoikis, and pyroptosis. Oncosis is a form of non-programmed cell death marked by cell swelling and karyolysis (6). Key factors leading to oncosis include increased cell volume, swelling of the nucleus and organelles, destruction of mitochondrial cristae, heightened cell membrane permeability, nuclear disintegration, and inflammatory responses.

Oncosis exerts its antitumor effects through two main mechanisms. Firstly, it directly induces the death of tumor cells. Secondly, it stimulates the release of inflammatory substances post-cell death, which recruits immune cells to enhance its antitumor activity. Although the precise mechanism by which oncosis directly causes cell death is not fully understood, studies suggest a link to ROS and mitochondrial dysfunction. For instance, Fluopsin C induces ROS accumulation and oncosis in MCF-7 and MD-MBA-231 cell lines (74). DHT has been shown to initiate porimin-dependent oncosis through ROS-mediated mitochondrial dysfunction in both *in vivo* and *in vitro* models, indicating its potential as a therapeutic agent for NSCLC (31). ROS are closely linked with mitochondrial function and are involved in downstream processes such as microvillar degradation and homotypic adhesion, which occur upstream of actin reorganization, plasma membrane damage, and mitochondrial membrane permeabilization (75). Porimin, a transmembrane protein, is part of the mucin family and acts as a receptor that mediates oncosis (76). When porimin binds to its ligand, it compromises the cell membrane integrity, leading to the formation of pores, increased membrane permeability, and eventually cell swelling and death. Oncosis is particularly noteworthy as an immunogenic type of cell death, offering a novel strategy for cancer treatment by inducing oncotic cell death and triggering robust antitumor immunity. This intense activation of oncosis results in the infiltration of numerous immune cells, which not only kills cancer cells but also activates immune responses to inhibit tumor growth. The antitumor immunity associated with oncosis is multifaceted. It begins with the release of DAMPs and inflammatory cytokines, which directly enhance the innate immune response and facilitate the recruitment of adaptive immune cells, increasing antigen presentation and leading to extensive immune activation. The inflammatory cytokine IL-1β promotes dendritic cell (DC) maturation, activates CD8^+^ T cells, and inhibits the differentiation of immunosuppressive regulatory T cells (77). IL-18 is crucial for natural killer cell recruitment and activation, as well as Th1 polarization (78). Together, these processes alter the immunosuppressive tumor microenvironment and increase the presence of tumor-infiltrating lymphocytes. Therefore, inducing acute and substantial oncosis in cancer cells represents a promising strategy for tumor treatment.

Traditional chemotherapeutic agents typically induce apoptosis in tumor cells. However, the emergence of drug resistance has limited the effectiveness of these treatments in recent years (79–81). Consequently, there is a pressing need to develop effective therapeutic alternatives. Oncosis presents a potential new strategy for treating non-small cell lung cancer (NSCLC), particularly in cases resistant to apoptosis. For instance, the cyclometalated Ir (III) complex can activate oncosis-specific proteins like porimin and calpain in the cisplatin-resistant A549R cell line, thereby inducing oncosis (32). Similarly, aspirin has been shown to induce oncosis in A549 cells by reducing Bcl-XL levels and depleting ATP (25). Ru3-M exhibited the ability to induce A549 oncosis by dramatically reducing intracellular ATP levels and destroying the membrane potential depolarization potential (82). Additionally, interleukin-33 has been found to enhance programmed oncosis in ST2L-positive low-metastatic cells within the lung cancer tumor microenvironment (83). Furthermore, arnicolide D (AD), a sesquiterpene lactone derived from centipeda minima, induces oncosis of hepatocellular carcinoma cells and inhibits tumor growth in xenograft-bearing nude mice. It has been shown that AD can induce endoplasmic reticulum stress, increase ROS production and upregulated oncosis-related protein, such as porimin (84). The advent of oncosis has led to a novel approach in the treatment of cancer. However, when oncosis occurs, the cell membrane ruptures, releasing intracellular substances and inducing inflammatory reactions. Consequently, future research should concentrate on the development of drug molecules that can induce tumor cells to undergo oncosis, thus providing a novel treatment option for tumors that are resistant to apoptosis.

## 8 Inducers of oncosis

Most natural products and small molecular compounds, whether currently available in clinical setting or under investigation as potential leads, act as cytotoxic agents that directly or indirectly destroy cancer cells and promote cell death. Oncosis is marked by energy depletion, failure of ionic pumps, cellular swelling, plasma membrane blebbing, mitochondrial swelling, and dilation of the endoplasmic reticulum. Research has identified various oncosis inducers, including naturally sourced substances, bacterial extracts, and synthetic compounds. Inducing oncosis might be a significant mechanism through which these agents exert their anticancer effects (**Table 2**).

**Table 2:** Summary of the representative inducers of oncosis.

Compounds	Structure	Cell line	Signaling pathway and mechanism	Relative diseases
Artesunate	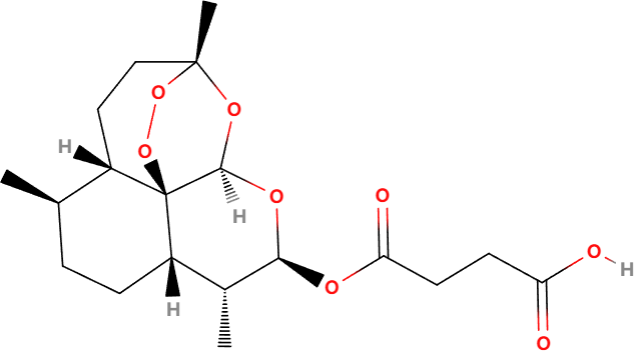	Panc-1, BxPC-3, CFPAC-1, SGC-7901, BGC-823, AGS	ROS accumulation; N-acetyl-cysteine accumulation; Ca^2+^ overload; upregulated calpain	PAAD/GC (24, 86)
Aspirin	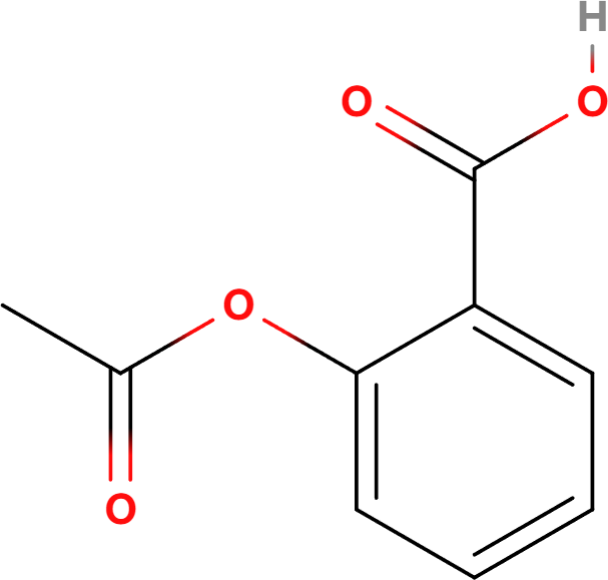	A549	ATP depletion; Bcl-XL inhibition	NSCLC (25)
Dihydrotanshinone	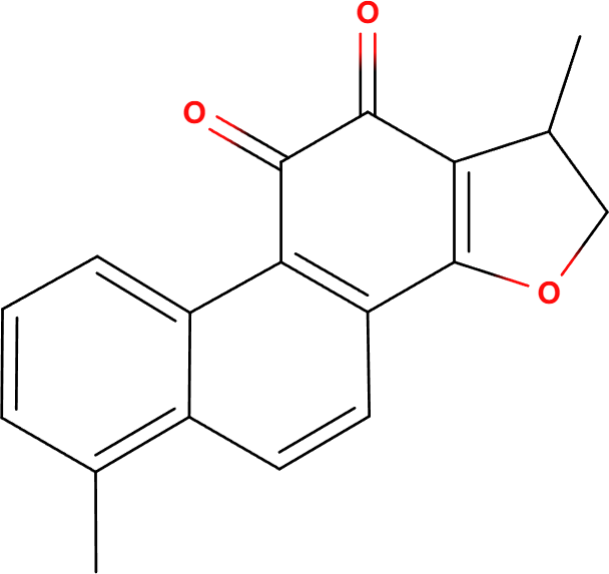	A549	Mitochondrial membrane permeability increase; intracellular ATP decrease; Cytoskeleton destroyed; induced porimin expression; ROS accumulation	NSCLC (31)
Fluopsin C	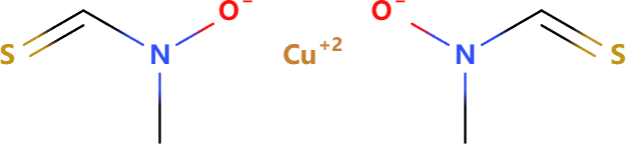	MCF-7, MDA-MB-231	ROS accumulation; cytoskeleton destroyed; Mitochondrial membrane permeability increase	BRCA (74)
Kahalalide F	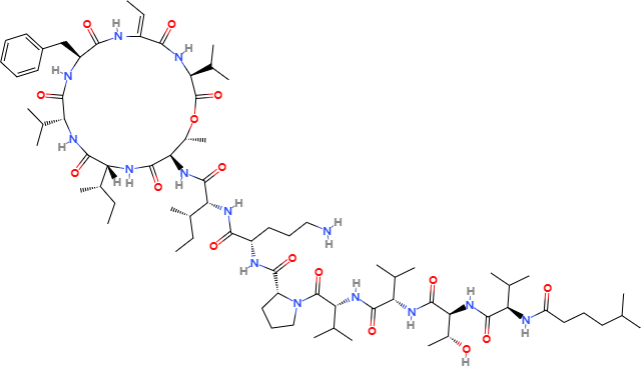	DU145, LNCaP, SKBR-3, BT474, MCF7	Mitochondrial membrane permeability increase; endoplasmic reticulum dilation	BRCA (88)
QC2	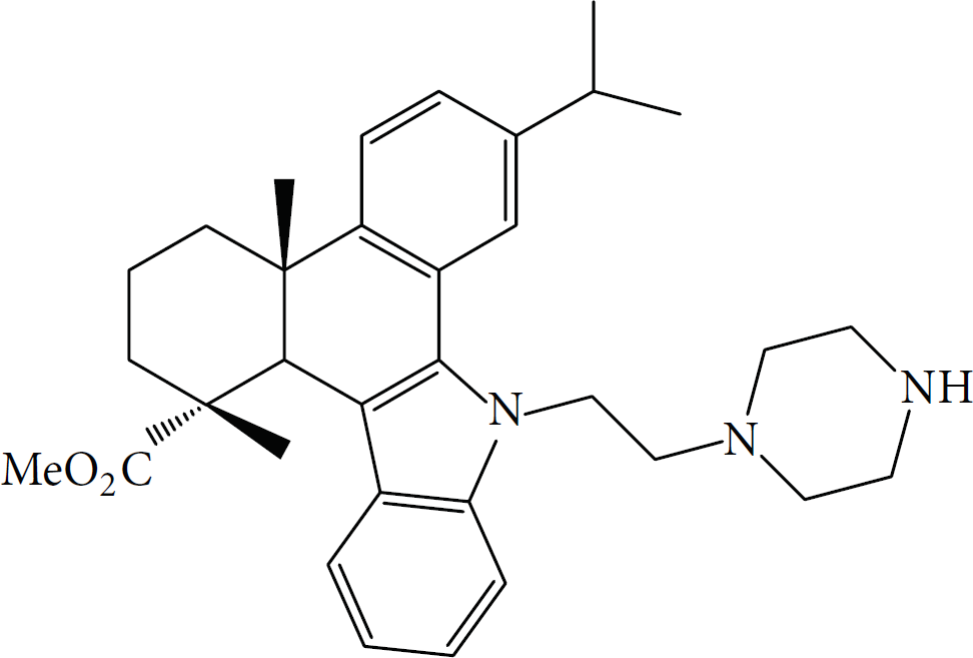	SMMC-7721	ROS accumulation, mitochondrial membrane permeability increase; cytoskeleton destroyed	LIHC (45)
QC4	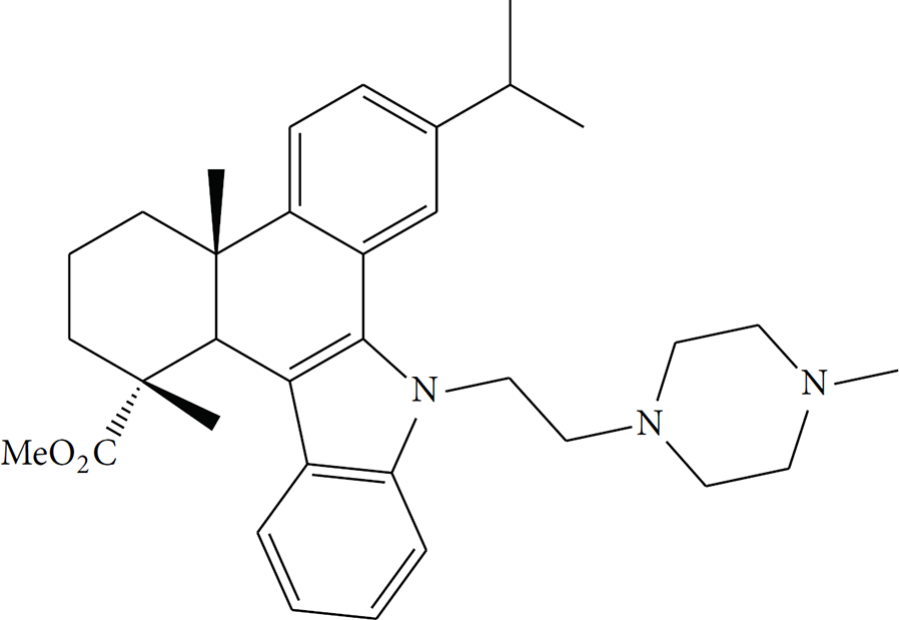	SGC-7901, MGC80-3	Activated calpain-1 autolysis; mitochondrial membrane permeability increase; ROS accumulation	GC (90)
Sanguinarine	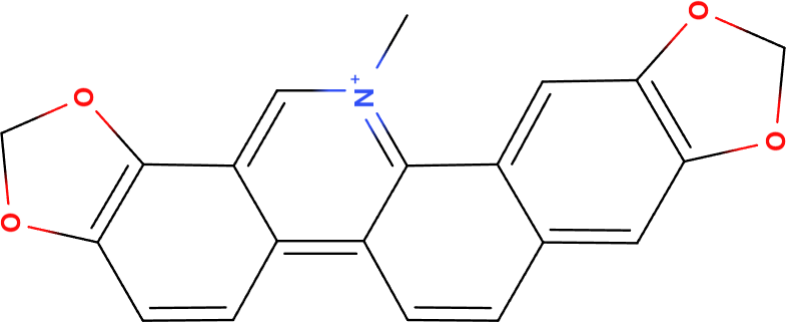	K562, KB, PC3, MCF-7	ROS accumulation	LAML (97)
Solamargine	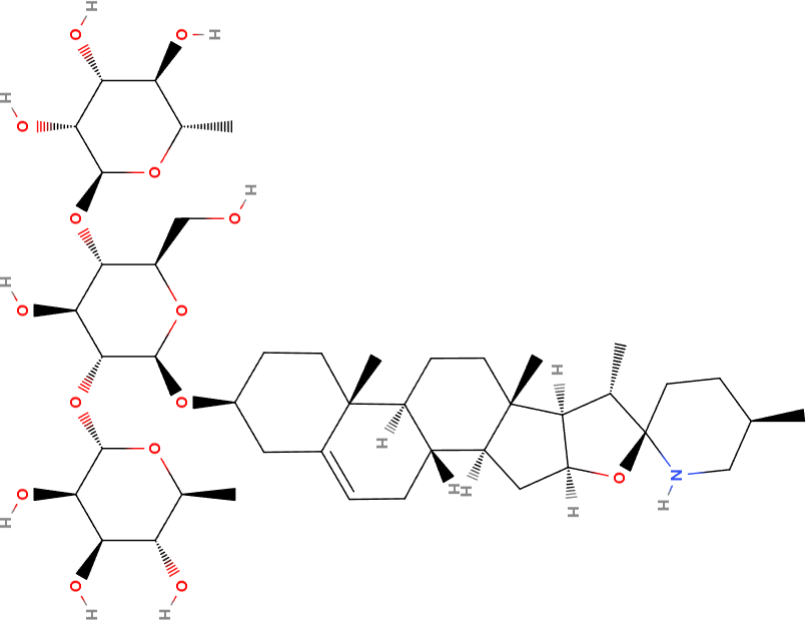	K562, KB	Cytoskeleton destroyed; intracellular ATP decrease; cellular membrane damaged	LAML/HNSC (92)

PAAD, Pancreatic adenocarcinoma; GC, Gastric cancer; NSCLC, Non-small cell lung cancer; BRCA, Breast invasive carcinoma; LIHC, Liver hepatocellular carcinoma; LAML, Acute myeloid leukemia; HNSC, Head and neck squamous cell carcinoma.

Artesunate (ART), derived from artemisinin, is the primary active ingredient in the Chinese medicinal herb *Artemisia annua* L (85). Artesunate is approved for treating multidrug-resistant malaria and has a strong safety profile (85). Studies have shown that ART selectively induces cytotoxic effects in human pancreatic cancer cells. Electron microscopy has revealed that ART triggers a cell death process characterized by significant swelling and various profound morphological changes, including the formation of numerous small and large cytoplasmic vacuoles, swollen and disorganized mitochondria, and nuclear dilation without chromatin condensation, eventually leading to cell lysis (86). ART-treated cells also show a loss of ΔΨm, and the cell death induced by ART can be inhibited by the ROS scavenger N-acetyl-cysteine (NAC). Moreover, *in vivo* studies using pancreatic cancer xenograft models have demonstrated that ART induces dose-dependent tumor regression, further supporting its anticancer activity through a form of oncosis-like cell death (86). Additionally, research by Zhou et al. showed that ART significantly inhibits the proliferation of gastric cancer cells and induces oncosis *in vitro*, while also mediating dose-dependent reductions in tumor size *in vivo* (86). Thus, ART holds promise as a therapeutic agent for pancreatic and gastric cancers.

Kahalalide F (KF), a cyclic depsipeptide with a long linear lipopeptide chain, was first isolated in 1993 from the *herbivorous mollusk Elysia rufescens* found in Blackpoint Bay, Hawaii (87). KF induces oncosis in human prostate and breast cancer cells and exhibits strong cytotoxic effects against both types of cancer cell (88). Cells treated with KF experience significant changes, including extensive cytoplasmic swelling and vacuolization, dilation and vesiculation of the endoplasmic reticulum, mitochondrial damage, and rupture of the plasma membrane. The cellular architecture is severely disrupted as soon as 1-3 hours after KF administration, compromising the integrity of essential organelles such as mitochondria, the endoplasmic reticulum, and lysosomes. Recent studies have shown that KF preferentially induces oncosis in tumor cells and is currently undergoing Phase II clinical trials.

Dehydroabietic acid (DHA) is a diterpene resin acid naturally derived from rosin, commonly found in coniferous trees, and has been investigated for its antimicrobial, anti-inflammatory, and anticancer properties (89). Among its derivatives, QC2 (45) and QC4 (90), have emerged as a promising candidates for their potent anticancer effects. The ATP levels of QC2-treated cells were found to be significantly reduced, with the depletion of ATP being regarded as the initial stage of oncosis (45). This process is characterized by destabilization of the cell membrane due to inactivation of ion pumps, mitochondrial dysfunction, and an increase in intracellular Ca^2+^, all of which exacerbate cellular damage and lead to oncotic death. These properties highlight the potential of QC2’s as a novel therapeutic option for hepatocellular carcinoma (45). In gastric cancer cells treated with QC4, notable morphological changes are observed, including plasma membrane blebbing, dilation of the endoplasmic reticulum, and mitochondrial swelling (90). Furthermore, QC4 administration induces both oncosis and apoptosis, indicating its ability to inhibit gastric cancer cell viability by triggering these two forms of cell death, positioning QC4 as a potential anticancer agent for gastric cancer treatment.

Dihydrotanshinone (DHT) is a bioactive compound from *Salvia miltiorrhiza* known for its anticancer properties against malignant tumors (31, 91). DHT has been found to induce porimin-dependent oncosis through ROS-mediated mitochondrial dysfunction, both *in vivo* and *in vitro*. These findings suggest that DHT could be a potential therapeutic agent for treating NSCLC. The oncosis induced by DHT is distinct from the apoptosis observed in NSCLC cells following DHT treatment. This distinction could be advantageous in the clinical chemotherapy of drug-resistant, apoptosis-resistant NSCLC cells (31).

Solamargine (SM), a steroidal alkaloidal saponin extracted from the herb *Solanum incanum*, effectively induces oncosis in human K562 leukemia and squamous cell carcinoma KB cells (92). SM-induced cell death is characterized by significant swelling and various profound morphological changes impacting various cytoplasmic organelles and the plasma membrane. During treatment with SM, microtubule networks were destroyed and short microtubule fragments were observed in the cytoplasm. SM also severely affects lysosomes and mitochondria, causing a loss of ΔΨm and lysosomal integrity. Interestingly, while SM can induce apoptosis at low doses, high doses can induce oncosis, with intermediate concentrations causing both types of cell death.

Aspirin, one of the most iconic drugs in medical history, is the most widely used nonsteroidal anti-inflammatory drug globally. Previous studies have demonstrated that aspirin possesses common anti-tumor properties, including the induction of apoptosis, autophagy, and anti-proliferative activity (93). It was found that aspirin induces oncosis, resulting in cell swelling and damage to the plasma membrane. In HeLa and A549 cells, aspirin induces oncosis by compromising the anti-apoptotic protein Bcl-XL and depleting intracellular ATP. Notably, Interestingly, this aspirin-induced oncosis occurs independently of caspase-3, a protease typically involved in apoptosis. Aspirin is more likely to induce oncosis in tumor cell lines, such as HeLa cells, which have low levels of both Bcl-XL and caspase-3. This suggests that aspirin could provide an alternative therapeutic approach for treating tumors.

Sanguinarine (SA) (94), a benzophenanthridine alkaloid extracted from the rhizomes of *Sanguinaria canadensis*, has been shown to be effective against multidrug resistance mediated by P-glycoprotein (95). Both P-gp-positive and negative cell lines demonstrated a notable apoptotic response at lower SA doses, while higher doses induced significant oncosis. The cell death induced by SA was characterized by oncosis, which manifested as cellular swelling and changes in membrane permeability. It is of note that SA demonstrated a minimal impact on normal human T lymphocytes. These findings suggest that SA possesses anticancer properties that can overcome drug resistance. Further research is needed to evaluate the potential of SA in reversing drug resistance and its application in cancer chemotherapy.

Fluopsin C, an antibiotic isolated from *Pseudomonas jinanesis*, *Pseudomonas* sp., *P. aeruginosa*, and *Streptomyces*, has demonstrated antitumor activity against various cancer cell lines (96).In breast cancer cells, Fluopsin C induces cell death characterized by swelling, formation of membrane surface blebs, loss of membrane integrity, distinct nuclear morphology, and release of LDH and cytoplasmic contents, all hallmark features of oncosis. Additionally, Fluopsin C leads to intracellular PI uptake, significantly increased LDH release, cytoskeletal degradation, ROS accumulation, decreased intracellular ATP levels, and loss of Δψm. Fluopsin C shows a time-dependent impact on the cytoskeleton, including the microfilament and microtubule networks, and affects the levels of β-Actin and α-Tubulin proteins (74). Despite these findings, the exact molecular mechanism by which Fluopsin C induces oncosis remains to be fully elucidated.

The induction of oncosis represents a novel direction of research that offers new insights into the anti-cancer mechanisms of these compounds. In the meantime, it is necessary to consider certain issues. However, there are important considerations to keep in mind. Oncosis inducers often act on multiple targets and can trigger several types of programmed cell death simultaneously. For example, artesunate can induce apoptosis, necroptosis, or ferroptosis in different cancer cells. A major concern is the pro-inflammatory response associated with oncosis. Unlike apoptosis, which minimally impacts inflammation, oncotic cell death leads to inflammation. Oncosis is characterized by rapid membrane permeabilization and rupture, resulting in the release of cellular contents, including DAMPs, and exposure to cytokines and chemokines. This pro-inflammatory nature is a significant challenge in this field. Finding ways to minimize inflammation while maintaining the antitumor effects is a critical direction for future research and development of oncosis inducers. Additionally, the potential cytotoxicity of oncosis inducers to normal cells, especially liver and kidney cells, must be carefully considered.

## 9 Method for detecting oncosis

Determining the specific type of cell death following cellular injury is crucial for diagnostic purposes, dose-response evaluations, and toxicological studies. Proper assessment and interpretation of cellular responses to severe injury, including both pre- and post-death changes, are vital as these changes can serve as early indicators of toxic reactions to various drugs, including those used in anticancer therapies.

Following signaling or damage-induced lesions, oncosis triggers a sequence of intracellular events that are characteristic of oncotic cell death. These include increased ROS production, mitochondrial swelling, ATP depletion, disruption of Ca^2+^ homeostasis, perinuclear clustering of organelles, activation of certain proteases like calpains and cathepsins, lysosomal rupture, and ultimately the rupture of the plasma membrane. Despite the clear delineation of these processes, the exact signals that initiate oncosis remain ambiguous. This ambiguity is partly due to the heterogeneous nature of oncosis, both in its causative factors and its pathological presentation, and the absence of a specific and versatile method for its detection beyond identifying ultrastructural features. To accurately identify the occurrence of oncosis, the following detection methods will be used based on the changing characteristics of cells, including membrane alterations, mitochondrial dysfunction, DNA fragmentation, ion imbalance, and mechanism-based assays.

### 9.1 Cell swelling detection

Oncosis, derived from the Greek word meaning “swell,” is a form of accidental cell death. Imaging techniques that detect oncosis typically focus on changes in membrane permeability and the appearance of ruptured blebs that create significant holes in the membrane. When examining cells under a light microscope, specific morphological alterations are evident, such as a substantial increase in cell volume, the formation of vesicular structures in the cell membrane, and cytoplasmic vacuolation. Using haematoxylin-eosin (H&E) staining (7), the nucleus becomes more visible under the microscope (98). It appears dark blue and purple, and the expanded cytoplasm appears pale pink. Additionally, obvious vacuoles can be observed in the cytoplasm and cell membrane. However, the resolution of light microscopes is limited, making it difficult to accurately capture even minor changes in cell structure, such as expansion of mitochondria and endoplasmic reticulum (70). Therefore, while the optical microscope is simple to operate and provides intuitive results, it only offers basic morphological change information on the process of oncosis. Further ultrastructural research requires the use of a microscope with higher resolution.

The transmission electron microscopy (TEM) can clearly display the ultrastructural changes in cells (98). Under the TEM, various changes can be observed, including swelling and clearance of cytoplasm, expansion of mitochondria and endoplasmic reticulum, dispersion of nuclear chromatin, and agglutination around the nuclear membrane and nucleolus (90). The electron microscope can reveal structural changes in oncosis from various angles, but it cannot capture the dynamic changes in cell morphology over time within the same field of view (70, 90). Considering the benefits and limitations of different microscopic techniques, light microscopy is a cost-effective and reliable method for detecting oncosis, although it lacks objectivity, reproducibility, and quantitative measurement, and is prone to errors that could be mitigated with higher magnification. For detailed electron microscopic analysis of oncosis, TEM is preferred over scanning electron microscopy (SEM), which primarily provides information on cell-cell interactions and surface features (7, 75). Oncosis is a fast-moving process characterized by significant morphological changes. Time-lapse microscopy (TLM) stands out as the most accurate technique for capturing the intricate dynamics and complexity of essential cellular processes at the level of individual cells, providing exceptional temporal precision. TLM allows for real-time observation and recording of morphological changes in cells over time. This method accurately captures changes in cell surface and shape, including swelling, vacuolization of the cytoplasm, and the formation of bubbles on the cell membrane. This technique provides dynamic detection and analysis of the development process of oncosis. TLM has advanced significantly in cell biology, helping to elucidate the dynamic complexities within cells. Furthermore, TLM shooting allows for the observation of the actual growth state and changes of cells without the need for cell labeling or destruction. This method is superior to ordinary optical microscope observation.

### 9.2 Cell membrane damage detection

Initially, cellular injury leads to the formation of membrane blebs. If the damaging stimulus continues, these blebs eventually rupture, resulting in cell lysis. Blebbing and subsequent membrane rupture are key indicators of oncotic cell death (99). During oncosis, cell membrane damage can be detected using impermeable dyes such as propidium iodide (PI), trypan blue, and 7-amino-actinomycin D (7-AAD). Unlike fluorescein diacetate, PI cannot penetrate intact cell membranes and only stains cells with compromised membranes, marking dead cells with a red fluorescence (100). These dyes are commonly used to distinguish between live and dead cells, especially when combined with Annexin V staining and either 7-AAD or PI staining (31, 101). This combination is effective for identifying apoptotic and oncotic cell death stages using flow cytometry. It should be noted, however, that Annexin V can bind to phosphatidylserines exposed on the inner membrane leaflet of necrotic cells, potentially yielding false-positive results (24, 102). To accurately distinguish apoptotic from oncotic cells, additional staining methods are required. One efficient approach involves a triple nucleic acid stain using Hoechst 33342, YO-PRO-1, and propidium iodide, which allows for flow cytometric identification of the total cell population (blue fluorescence), apoptotic cells (green fluorescence), and dead cells (red fluorescence) (62).

Lactate dehydrogenase (LDH) is a tetrameric enzyme composed of two distinct types of 35 kDa subunits. LDH is found in all cells but is released into the extracellular environment only when the plasma membrane is damaged (103). The damage to the cell membrane leads to the leakage of intracellular contents, including LDH, into the surrounding medium. Measuring the concentration of LDH in the supernatant collected from cell cultures provides an indirect assessment of the extent of oncosis. The supernatant was collected from the cell culture and the degree of oncosis was indirectly measured using the LDH leakage assay (74).

### 9.3 Mitochondrial dysfunction detection

Mitochondrial dysfunction plays a pivotal role in initiating oncotic cell death. As the primary source of cellular ATP, mitochondria produce over 90% of the cell’s ATP. When mitochondrial function is impaired, there is a substantial reduction in ATP production. This depletion of ATP disrupts the normal flux of ions and water across cell membranes, resulting in cell swelling and advancing the process of oncosis. Furthermore, mitochondrial dysfunction enhances the production of ROS and compromises Ca^2+^ regulation (75). The Ca^2+^ overload triggers the mitochondrial permeability transition, causing inner compartment swelling.

MPTP is a non-specific and Ca^2+^-dependent channel composed of inner and outer membrane components of mitochondria. The depolarization of the ΔΨm leads to the opening of membrane channel holes, which significantly alters the permeability of mitochondria and causes them to swell. Calcein AM, a fluorescent probe of membrane permeability, enters cells and accumulates in cytoplasm and mitochondria. Calcein AM is a non-fluorescent compound that enters the cell. Once inside, it is hydrolyzed by esterase to remove acetyl methyl ester. This results in the formation of a polar fluorescent dye called calcein, which is unable to permeate the cell membrane (101). This conversion leads to a strong green fluorescence in the cytoplasm and mitochondria. To specifically assess mitochondrial integrity, cobalt chloride (CoCl_2_) is used to quench the green fluorescence of calcein in the cytoplasm. Normally, CoCl_2_ cannot enter mitochondria because the MPTP of mitochondria is closed (104). Thus, in healthy mitochondria, the green fluorescence from calcein remains in the mitochondria. However, when mitochondrial damage occurs, the opening of MPTP, allowing CoCl_2_ to enter mitochondria. This eventually weakens or eliminates calcein green fluorescence in mitochondria. The level of mitochondrial MPTP opening can be determined by the intensity of calcein green fluorescence within the mitochondria (105). A stronger green fluorescence indicates a lower degree of opening, while a weaker green fluorescence indicates a higher degree of opening.

Mitochondrial membrane potential (ΔψM) is an important indicator of mitochondrial function and cell death. The opening of transition pores in the inner mitochondrial membrane results in a reduced transmembrane potential, release of cytochrome c into the cytosol (32). The ΔΨm is suitable for quantifying changes in live cells (106). Cells are often stained with ΔψM-sensitive dyes, such as rhodamine 123 or the more recently used JC-1 dye (5,5’,6,6’-tetrachloro-1,1’,3,3’-tetraethylbenzimidazolylcarbocyanine iodide), which accumulates in active mitochondria. Among ΔψM assays, JC-1 is particularly favored for monitoring mitochondrial damage and cell death (31). The dye’s accumulation in mitochondria causes a shift in fluorescence from green to red as JC-1 aggregates form. Mitochondrial depolarization is indicated by a decrease in the red/green fluorescence ratio (32).

In recent years, many studies have reported a relationship between oncosis and overproduction of ROS. For example, artesunate has been shown to induce pancreatic oncosis through ROS (75). ROS levels are often measured using DCFH-DA (2’,7’-dichlorofluorescin diacetate), a non-fluorescent probe that permeates cells. Once oxidized by ROS, DCFH-DA is converted into the fluorescent compound DCF (2,7-dichlorofluorescein), which can be quantified through flow cytometry. The fluorescence intensity of DCF can be used to detect the level of ROS in cells, which may indicate cell death (24).

Earlier assessments of mitochondrial respiration and dysfunction focused on mitochondrial respiratory control, specifically the increase in respiration rate in response to ADP, measured through oxygen consumption in mitochondrial preparations. Oxygen consumption was typically measured using polarographic methods, such as Clark-type oxygen electrodes (82), and various chemicals that affect mitochondrial respiration, including uncouplers, oligomycin, and atractyloside. For intact cells, the most informative assay involves measuring cell respiratory control, which includes the rate of ATP production, proton leak rate, coupling efficiency, maximum respiratory rate, respiratory control ratio, and spare respiratory capacity (107). The level of ATP depletion in cells is a crucial factor in distinguishing between oncosis and apoptosis. Cells undergo apoptosis when the ATP level is depleted by 25-70%, whereas oncosis is initiated when the depletion level reaches 70-85%. Mitochondrial ATP synthesis in cultured mammalian cells can be measured by permeabilizing the plasma membranes with streptolysin O without harming mitochondrial function. ATP synthesis is then monitored using a luciferin/luciferase-based bioluminescence assay and quantified with a microplate luminometer (108).

### 9.4 Ion imbalance detection

During the prophase of oncosis, disruption of Ca^2+^-ATPase and Na^+^-K^+^-ATPase on the cell membrane leads to an influx of Ca^2+^ and an efflux of K^+^. The cell membrane permeable K^+^ indicator ION Potassium Green-2 AM can be used to detect the level of potassium ions in cells and reflect potassium ion efflux. For Ca^2+^ detection, fluorescent probes are widely employed, categorized based on the wavelengths, including visible light excited Ca^2+^ fluorescent probes (e.g. Fluo-3AM, and its upgraded version Fluo-4AM, as well as Rhod-2) and ultraviolet light excited Ca^2+^ fluorescent probes (e.g. Indo-1 and Fura-2). Fluo-4AM, in particular, loads faster and has stronger detection capabilities compared to Fluo-3AM at the same concentration. In the field of Ca^2+^ concentration research, fluorescent probes are commonly used to observe changes in intracellular Ca^2+^ levels through specific binding with Ca^2+^. Upon binding with Ca^2+^, these probes exhibit a significant increase in fluorescence intensity, which can be detected using fluorescence microscope and flow cytometry (109).

### 9.5 Nuclear changes detection

In the early stages of oncosis, nuclear chromatin clumps along the nuclear envelope and around the nucleolus, resulting in clarification of the remaining nucleoplasm. For detecting apoptosis, DNA ladder assay is a cost-effective and straightforward method that does not require commercial kits or special equipment. This method leverages the characterized DNA fragmentation pattern seen during apoptosis. Apoptosis induces DNA laddering due to the cleavage of internucleosomal linker regions by caspase-activated DNase (CAD), resulting in the generation of multiple nucleosome units of 180-185 DNA base pairs (110). To conduct a DNA ladder assay, apoptotic cells are lysed, and their DNA is extracted using chloroform-isoamyl alcohol, followed by isopropanol. The extracted DNA fragments are then separated on a 1.5% agarose gel and visualized with ethidium bromide staining, which reveals a characteristic ladder-like pattern (110). In contrast, during oncosis, DNA is broken down into diffuse and random fragments. Electrophoretic analysis of these fragments displays random electrophoresis bands rather than the distinct pattern in apoptosis. It is important to note that other forms of cell death may also exhibit similarities to oncosis (21).

In addition to the DNA ladder assay, the TdT-dUTP nick end labeling (TUNEL) method is widely used to detect DNA fragmentation in apoptotic cells (111). However, methods based on nick translation are not exclusive to apoptosis, as they can also detect DNA breaks in necrotic cells. The TUNEL assay by incorporating nucleoside triphosphates into DNA strand breaks and then labelled these larger nucleotides with a marker (such as biotin or digoxigenin), which can’t penetrate intact cells. One significant advantage of the TUNEL assay is its ability to detect DNA strand breaks in the early stages of apoptosis, even before any morphological changes are visible.

### 9.6 Cytoskeleton changes detection

Morphologically, the formation of blebs is associated with significant alterations in the cytoskeleton, affecting both tubulin and actin. ATP depletion profoundly impacts the structure and function of the cytoskeleton, mainly causing the severing or fragmentation of F-actin filaments during ATP depletion (112). To study these changes, fluorescent probes such as tubulin-Tracker and actin-Tracker, are used to stain the cytoskeleton. In normal cells, both probes stained the cytoskeleton evenly, indicating a complete and orderly distribution of the cytoskeleton throughout the cytoplasm. However, when cells undergo oncosis, the cytoskeleton is destroyed, and its integrity is lost. In addition to immunofluorescence, western blotting can also be employed to detect protein degradation of actin and tubulin (75, 113). Given the substantial changes in cytoskeletal proteins during oncosis, it is recommended that these proteins should not be used as reference proteins (loading control) in the study of oncosis.

### 9.7 Detection of oncosis related proteins

Western blotting, qPCR and immunofluorescence were used to detect the expression of porimin and calpain. Porimin is often regarded as a key marker for identifying oncosis due to its specific expression on the cell membrane (114). The presence of porimin on the cell surface is crucial for confirming the occurrence of oncosis. Calpain, another crucial indicator of oncosis, exhibited a markedly elevated expression level in cells undergoing oncosis (42, 44). A growing number of drugs have been discovered to induce oncosis, making it an area that warrants thorough investigation. Currently, no standard method exists for detecting oncosis. Largely because it shares morphological and biochemical similarities with other forms of cell death, making it difficult to detect using a single method. Therefore, the use of multiple methods in combination is necessary to confirm its occurrence. To differentiate oncosis from other cell death pathways, the effect of various inhibitors, including pan-caspase inhibitor Z-VAD-FMK, autophagy inhibitor 3-MA, necroptosis inhibitor Necrostatin-1, pyroptosis inhibitor MCC950, and ferroptosis inhibitor Ferrostatin-1, on drug-induced cell death was investigated. By applying these inhibitors, we were able to exclude pathways such as apoptosis, autophagy, necrosis, pyroptosis, and ferroptosis. Future research should focus on developing more accurate and specific detection methods for oncosis. This will facilitate systematic and in-depth studies into the role of oncosis in the occurrence and development of tumors, exploration of potential anti-tumor targets, and investigation of the pathological mechanism of oncosis in other diseases.

## 10 Conclusion and perspectives

Cell death is crucial for numerous biological processes, including maintaining tissue equilibrium, organ development, immune function, inflammatory responses, and various pathological conditions. Programmed cell death has evolved into different forms to remove damaged or infected cells, allowing healthy cells to function optimally. These forms include energy-dependent processes like apoptosis, autophagy, and pyroptosis, and energy-independent processes such as oncosis and necrosis. Each type exhibits unique morphological and biochemical characteristics and can be classified as either non-inflammatory or inflammatory. Apoptosis, a non-inflammatory process, removes damaged cells in an orderly manner, avoiding harm to neighboring tissues. In contrast, oncosis, necrosis, and pyroptosis trigger inflammatory responses, potentially causing damage to surrounding cells (115).

Although a plethora of techniques exist for the detection of oncosis, their specificity is often limited. Therefore, combining two or more techniques is recommended to distinguish apoptotic from oncotic cells. As our understanding of oncosis deepens, the complexity of studying cell death increases. This is especially true because the molecular mechanisms of oncosis are interwoven with those of other cell death forms, potentially leading to their co-activation. The unique molecular markers used to distinguish oncosis from other types of cell death are becoming less clear. It is evident that several cell death cascades coexist and interact at multiple levels, functioning in a synergistic, simultaneous, or consecutive manner within the same cell. While apoptosis, pyroptosis, and necroptosis are distinct cell death pathways, they collectively constitute a single, synchronized and coordinated cell death system, that allows for compensation interactions. This implies that cell death does not occur via a single, isolated pathway; instead, there is a degree of interaction between the pathways, with one pathway exerting a profound influence on the activity of another.

Oncosis is a form of inflammatory cell death characterized by cell swelling, mitochondrial enlargement, endoplasmic reticulum dilation, nuclear chromatin clumping, and rupture of the plasma membrane (6). As oncosis progresses, the depletion of intracellular energy stores leads to the failure of plasma membrane ionic pumps (6). This failure causes cellular debris to leak into surrounding tissues, triggering inflammation and damaging neighboring cells. Oncosis is particularly evident in models of ischemic injury and can lead to oncotic necrosis (116). Unlike apoptosis, which involves cell shrinkage, chromatin margination in the nucleus, and the formation of apoptotic bodies, oncotic necrosis is characterized by cell swelling, karyolysis, vacuolation, and lysis, followed by the release of cellular contents (21). The processes of oncosis and apoptosis are interconnected through their dependence on ATP. Notably, a cell undergoing apoptosis may deplete its ATP supply, preventing it from completing apoptosis and leading to secondary necrosis, characterized by swelling and lysis. Conversely, inhibiting oncosis in stressed cells can induce apoptosis (21). The transition from apoptosis to secondary necrosis via oncosis indicates that apoptosis and oncosis are not entirely separate mechanisms but represent two extremes of necroptosis. This is supported by the observation that the opening of the MPTP occurs in both oncosis and apoptosis (117). The hypothesis suggests that minor mitochondrial damage can be repaired through autophagy of damaged organelles. However, extensive mitochondrial damage and high levels of cytochrome c release will activate the intrinsic apoptosis pathway if sufficient ATP is available. Severe damage with insufficient ATP will result in oncotic necrosis. Thus, oncosis may serve as a potential link between autophagy, apoptosis, and necrosis.

Cell death is undeniably beneficial in certain contexts, such as in cancer treatment. However, a major challenge in treating cancer is that cancer cells can develop resistance to various chemotherapeutic agents and evade the most common cell death pathway, apoptosis. Cancer cells are highly heterogeneous, showing different levels of drug sensitivity, resistance to treatment, and induction of various cell death forms. Therefore, it is crucial to explore and identify alternative methods for inducing different cell death pathways in cancer therapy. This review focuses on cell death pathways, particularly oncosis, which can be activated in cells resistant to chemotherapy due to inhibition of apoptosis, offering a promising therapeutic approach.

Oncosis differs from apoptosis in both its morphological changes and its underlying mechanisms, making it a significant target for addressing drug resistance in cancer therapy. Oncosis promotes the inflammatory death of cancer cells and inhibits their proliferation and migration. Unfortunately, the expression of genes associated with oncosis is often decreased or down-regulated in cancer cells. Despite being a concept recognized earlier than apoptosis, there has been less research focused on discovering drugs that initiate oncosis compared to those that induce apoptosis or other types of programmed cell death. This review highlights various natural and small-molecule compounds that can induce oncosis, their modulators, and the markers up-regulated during oncosis (e.g., porimin, calpain), as well as the roles of ion channels like K^+^ and Ca^2+^. Although many compounds have been identified that induce or modulate oncosis and have strong anti-tumor effects, no specific inhibitors of oncosis have been developed yet. Future research should aim to develop more specific inhibitors and to modulate key genes involved in oncosis regulation. By comparison, caspase-3 inhibitors like Ac-DEVD-CMK and DNA fragmentation detection have been commonly used for identifying and characterizing apoptosis (118). One of the most intriguing challenges in oncosis research is the lack of effective inhibitors and markers for the characterization of this cell death pathway. The recent insights into the crosstalk between apoptosis and oncosis provides a prospective framework for further research into the potential connections between different death pathways and their effects on each other.

In summary, our review suggests that strategies for the pharmacological modulation of novel tumor cell death pathways could be highly beneficial in cancer treatment. We advocate for future studies utilizing animal models to explore these pathways further. Research focused on understanding the mechanisms of oncosis will deepen our knowledge of its role in cancer growth and proliferation and aid in developing more effective anticancer drugs targeting molecules involved in oncosis. Additionally, clinical trials to assess the potential benefits of modulating oncotic cell death in cancer patients are encouraged.
